# The "Universal Review" for April

**Published:** 1889-05-11

**Authors:** 


### THE "UNIVERSAL REVIEW" FOR APRIL.

The last number of the *Universal* is weak where it is wont to be strongest. The illustrations are much inferior to those Mr. Quilter has given us before and has taught us to look for. Those intended for the explication of "L'Amour Sublime" are the worst—fearful and wonderful smudges they are; but perhaps they are good enough for the letter-press, for the author, le Comte de Villiers de l'Isle Adam, has, in Western phrase, "played it low down" on the British public. He knows that many of us profess a liking for French stories and a comprehension of them beyond what we possess; and, trading on our ignorance, he has assumed that we would take twaddle for literature if only it was written in French. Yet the story of M. Rousseau-Latouche, his unjust suspicions and his mean mode of verifying them, might in the hands of an artist have been made into a satire that would touch the fountains of either laughter or tears; but as it is, we get something neither comic nor tragic, only unnecessary.

Of the three notices of John Bright, the brief note by Mr. Villiers is undoubtedly the most pleasing. In his longer tribute Professor Thorold Rogers is hampered by the fact that he is speaking of one who, though a personal friend, was at the last a political opponent. Mr. Quilter's allusion to the late politician takes the popular estimate of him too lightly. To say that "he appealed to the popular imagination in somewhat the same fashion as a strong, good-humoured dragoon of fiction," is to ignore the fact that he appealed to the highest side of the Puritan instincts of the English character. All the more, perhaps, because he belonged to a sect that calls no man master, it was felt that "he reverenced his conscience as his king"; even those who differed from him most did not dare to hint that any of his actions were due to self-seeking or the weakness that prompts to compromise. Mr. Quilter admits this when he says, "No man of equal rank has in this modern day shown himself so superior to the temptations of ambition and the promptings of personal interest." It is this, more than his power of oratory, or even his achievements in the cause of reasonable freedom, which has caused Mr. Bright's death to be so generally mourned, as the loss of one of the most important forces that make for political morality.

Mr. Quilter's article on English art is very depressing in tone, but a nation that never goes to see the works of its greatest artists, to whom Turner, Cox, and De Witt are only names, must expect reproaches. Two articles are dedicated to literature. The first of these, by C. S. Greene, deals with Australian writers, that little band who essay to serve the Muses beneath the Southern Cross. With the name of Adam Lindsay Gordon, if not with his work, English readers are familiar. It is one of the most marked instances extant, in which a strong individuality has shone out through imperfections of art and appropriation of others' styles. But very few know of Marcus Clarke, whose novel, "For the Term of His Natural Life," must be, if the quotations Mr. Greene gives be a fair sample of the whole, a book that English literature should be proud to claim. The suicide of the two child convicts is such a pitiful incident that one feels horror-stricken that it should be founded on fact. A friend of the boys, "Cranky Brown," had "done it"—that is, thrown himself from the cliff into the sea, and when they are going to look at him "to see if he looked happy," they meet Sylvia, the wife of the inspector, who is touched by the misery of their fate. But the narrative goes on:

> "Unfortunately, when Sylvia went away, Tommy and Billy put into execution a plan which they had carried in their poor little heads for some weeks.
> "'I can do it now,' said Tommy. 'I feel strong.'
> "'Will it hurt much, Tommy?' said Billy, who was not so courageous.
> "'Not so much as a whipping.'
> "'I'm afraid! Oh, Tom, it's so deep! Don't leave me, Tom!'
> "The bigger boy took his little handkerchief from his neck, and with it bound his own left hand to his companion's right.
> "'Now, I *can't* leave you.'
> "'What was it the lady that kissed me said, Tommy?'
> "'Lord have pity on them two fatherless children,' repeated Tommy.
> "'Let's say it, Tom!'
> "And so the two babies knelt on the brink of the cliff, and raising the bound hands together, looked up at the sky, and ungrammatically said, 'Lord, have pity of we two fatherless children!' and then they kissed each other and did it."

The other literary article is a eulogy of Hendrik Ibsen. Mr. Symons, who writes it, dwells on Ibsen's power of "seeing life steadily and seeing it whole." Perhaps this is the praise Ibsen least deserves. His work is powerful, and within limits versatile, but the spirit that permeates all is that of pessimism, and pessimism, as much as its opposite, is possible only to a man who sees but a part of life. As a literary force, Ibsen approaches Tolstoi, who certainly does not shirk depicting the life of our time and the faults of our society; but the Russian writer keeps himself in touch with the bright and honest side of human nature in a way the Scandinavian seems to be incapable of.

Mr. L. M. Gattie's revelations about "Life Insurance in 1889," and Mr. Burdett's indictments of those who hold "John Bull's Purse-Strings" are calculated to make the average mortal more thankful than he is wont to be that his pecuniary responsibilities are light; and Mr. George Kilgour's narrative of a journey from Kimberley to Delagoa Bay is very pleasant readiug.

